# Structure and plasticity of silent synapses in developing hippocampal neurons visualized by super-resolution imaging

**DOI:** 10.1038/s41421-019-0139-1

**Published:** 2020-02-25

**Authors:** Cheng Xu, Hui-Jing Liu, Lei Qi, Chang-Lu Tao, Yu-Jian Wang, Zeyu Shen, Chong-Li Tian, Pak-Ming Lau, Guo-Qiang Bi

**Affiliations:** 1 Hefei National Laboratory for Physical Sciences at the Microscale, University of Science and Technology of China, Hefei, Anhui 230027 China; 2 School of Life Sciences, University of Science and Technology of China, Hefei, Anhui 230027 China; 3 CAS Key Laboratory of Brain Function and Disease, University of Science and Technology of China, Hefei, 230027 China; 4 CAS Center for Excellence in Brain Science and Intelligence Technology, and Innovation Center for Cell Signaling Network, University of Science and Technology of China, Hefei, Anhui 230027 China

**Keywords:** Super-resolution microscopy, Membrane trafficking, Super-resolution microscopy, Membrane trafficking

## Abstract

Excitatory synapses in the mammalian brain exhibit diverse functional properties in transmission and plasticity. Directly visualizing the structural correlates of such functional heterogeneity is often hindered by the diffraction-limited resolution of conventional optical imaging techniques. Here, we used super-resolution stochastic optical reconstruction microscopy (STORM) to resolve structurally distinct excitatory synapses formed on dendritic shafts and spines. The majority of these shaft synapses contained *N*-methyl-d-aspartate receptors (NMDARs) but not α-amino-3-hydroxy-5-methyl-4-isoxazolepropionic acid receptors (AMPARs), suggesting that they were functionally silent. During development, as more spine synapses formed with increasing sizes and expression of AMPARs and NMDARs, shaft synapses exhibited moderate reduction in density with largely unchanged sizes and receptor expression. Furthermore, upon glycine stimulation to induce chemical long-term potentiation (cLTP), the previously silent shaft synapses became functional shaft synapses by recruiting more AMPARs than did spine synapses. Thus, silent shaft synapse may represent a synaptic state in developing neurons with enhanced capacity of activity-dependent potentiation.

## Introduction

In the mammalian brain, excitatory communication between neurons is primarily mediated by glutamatergic synapses^1,2^. Activity-induced plasticity of these synapses is believed to underlie learning and memory function of the brain^3–6^. Electrophysiological studies have suggested that excitatory synapses may exhibit distinct functional properties or states^7,8^. An extreme case is the so-called silent synapse^9–12^, which contains few α-amino-3-hydroxy-5-methyl-4-isoxazolepropionic acid receptors (AMPARs) and cannot carry out excitatory transmission upon presynaptic activation, but can be converted into the functional form through activity-dependent plasticity^13–16^. However, the structural and morphological correlates of these functional states have been lacking. Studies with electron microscopy (EM) have indicated that most glutamatergic excitatory synapses are formed on dendritic spines, in contrast to GABAergic inhibitory synapses that are primarily formed on dendritic shafts, although exceptions have been observed that some excitatory synapses formed directly on the shafts^17–20^. With conventional fluorescence microscopy, it was observed that early in development, N-Methyl-D-aspartate receptors (NMDARs) clusters might form on dendritic shafts before clustering of AMPARs^21^. Unfortunately, the diffraction-limited resolution of conventional optical microscopy does not allow for unambiguous determination whether these receptor clusters are actual shaft synapses. Thus, a higher-resolution imaging approach is desired to establish the link between the morphological and functional states of these synapses. In the current study, we took advantage of single molecule localization-based super-resolution fluorescence microscopy^22,23^ and its quantitative capability, to investigate in cultured hippocampal neurons the morphology and receptor expression of different forms of excitatory synapses and their changes during development and plasticity.

## Results

**Fig. 1:**
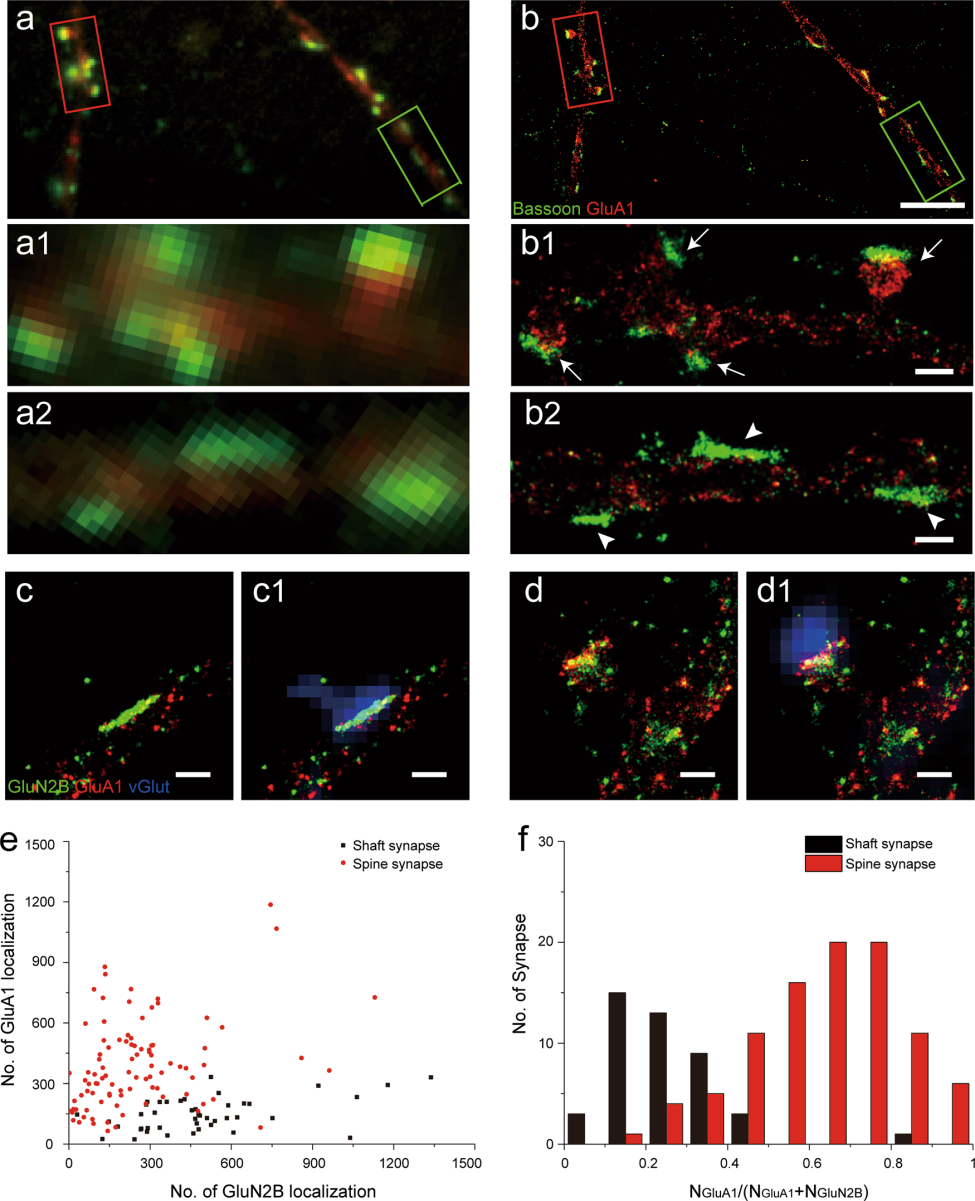
Excitatory shaft and spine synapses revealed by STORM imaging. **a, b** Fluorescence microscopy of cultured hippocampal synapses with immunostaining of bassoon (green) and postsynaptic GluA1 (red). Compared to conventional imaging (**a**), STORM imaging (**b**) shows better differentiation of two synaptic morphologies: spine synapse, indicated by arrow in (**b1**), and shaft synapse, indicated by arrow head in (**b2**). Scale bars, (**b**): 5 µm; (**b1**, **b2**): 500 nm. **c**, **d** STORM images of shaft (**c**) and spine (**d**) synapses with immunostaining of GluN2B (green), GluA1 (red), combined with conventional fluorescence images (**c1**, **d1**) of excitatory presynaptic maker vGlut1 (blue). Scale bars: 500 nm. **e** Scatter plot of GluN2B and GluA1 localizations in shaft (black) and spine (red) synapses. Note that the localization number refers to the measure number of single molecule blinking event, and is much larger than the actual number of receptors (see Methods). **f** Histogram of *N*_GluA1_/(*N*_GluA1_ + *N*_GluN2B_) in shaft (black) and spine (red) synapses. *n* = 44 (shaft), 94 (spine).

In the current study, we used low density culture of rat hippocampal neurons that formed synaptic connections starting from ~11 days in vitro (DIV). With immunofluorescence labeling of presynaptic scaffolding protein bassoon and postsynaptic AMPARs subunit GluA1, many synapses were visible under conventional fluorescence microscopy as fluorescent puncta with overlapping bassoon and GluA1 signals and without much discernable substructures (Fig. 1), because these synapses were usually hundreds of nanometers in size, close to the diffraction limit of optical microscopy. Thus, it is often hard to determine whether a fluorescent punctum near the dendrite is really a short spine synapse or a shaft synapse (see also Supplementary Fig. S1). Super-resolution stochastic optical reconstruction microscopy (STORM)^24,25^ with >10-fold improvement in resolution (Supplementary Fig. S2), has allowed for visualization of finer structural details of these synapses (Fig. 1b). Importantly, with STORM resolution dendritic GluA1 distribution facilitated visualizing dendritic profiles (Supplementary Fig. S3), it became much easier to determine whether a synapse was formed on the spine or dendritic shaft (Fig. 1b1, b2 and Supplementary Fig. S1d–f). From the STORM images, it was clear that a spine synapse generally contains postsynaptic AMPARs to oppose the presynaptic bassoon localizations. In contrast, most shaft synapses contained few AMPARs to oppose bassoon localizations (Fig. 1b2), although this was often hard to resolve in the conventional images.

To determine whether these AMPARs-negative shaft synapses were excitatory silent synapses, we performed STORM imaging of NMDARs and AMPARs using antibodies against the 2B subunit of NMDARs (GluN2B) and GluA1 containing-AMPARs, respectively, in conjunction with conventional immunofluorescence imaging of vesicular glutamate transporter 1 (vGlut1). Under STORM resolution, many GluN2B positive but GluA1 negative puncta were observed with distinct line-shaped structure formed directly along the dendritic shaft (Fig. 1c). Furthermore, virtually all such line-shaped puncta on the shaft were also co-localized with vGlut1 puncta similar to the excitatory spine synapses that contained both GluN2B and GluA1 (Fig. 1c1, d1 and Supplementary Fig. S4), indicating that they were indeed excitatory synapses. However, because of the lack of GluA1-containing receptors that are the dominant AMPARs in hippocampal synapses^26,27^, these shaft synapses were most likely to be functionally silent.

With STORM imaging, we were able to assess the expression of AMPARs and NMDARs using the number of single molecule localizations as a quantitative measure (see Methods)^28^. Figure 1e, f summarizes the localization numbers of GluN2B and GluA1 for all putative excitatory synapses identified by vGlut1 puncta from DIV 17 cultures. It is clear that most shaft synapses had low AMPAR proportion (defined as *N*_GluA1_/(*N*_GluA1_ + *N*_GluN2B_), see Methods) and could be classified as “silent synapses”, in contrast to the majority of spine synapses that belonged to the class of “functional” synapses with higher AMPAR proportion (Fig. 1e, f and Supplementary Fig. S5). Notably, there also existed a relatively small number of spine-shaped silent synapses, consistent with previous observations using conventional immunofluorescence imaging^21^. With 3D STORM, we also observed that for the silent shaft synapses, GluN2B localizations appeared to be primarily on or near the cell surface (Supplementary Fig. S6a and Supplementary Movies S1). Similar surface expression pattern was also found for GluN2B and GluA1 localizations in dendritic spines (Supplementary Fig. S6b-d and Supplementary Movies S2).

**Fig. 2:**
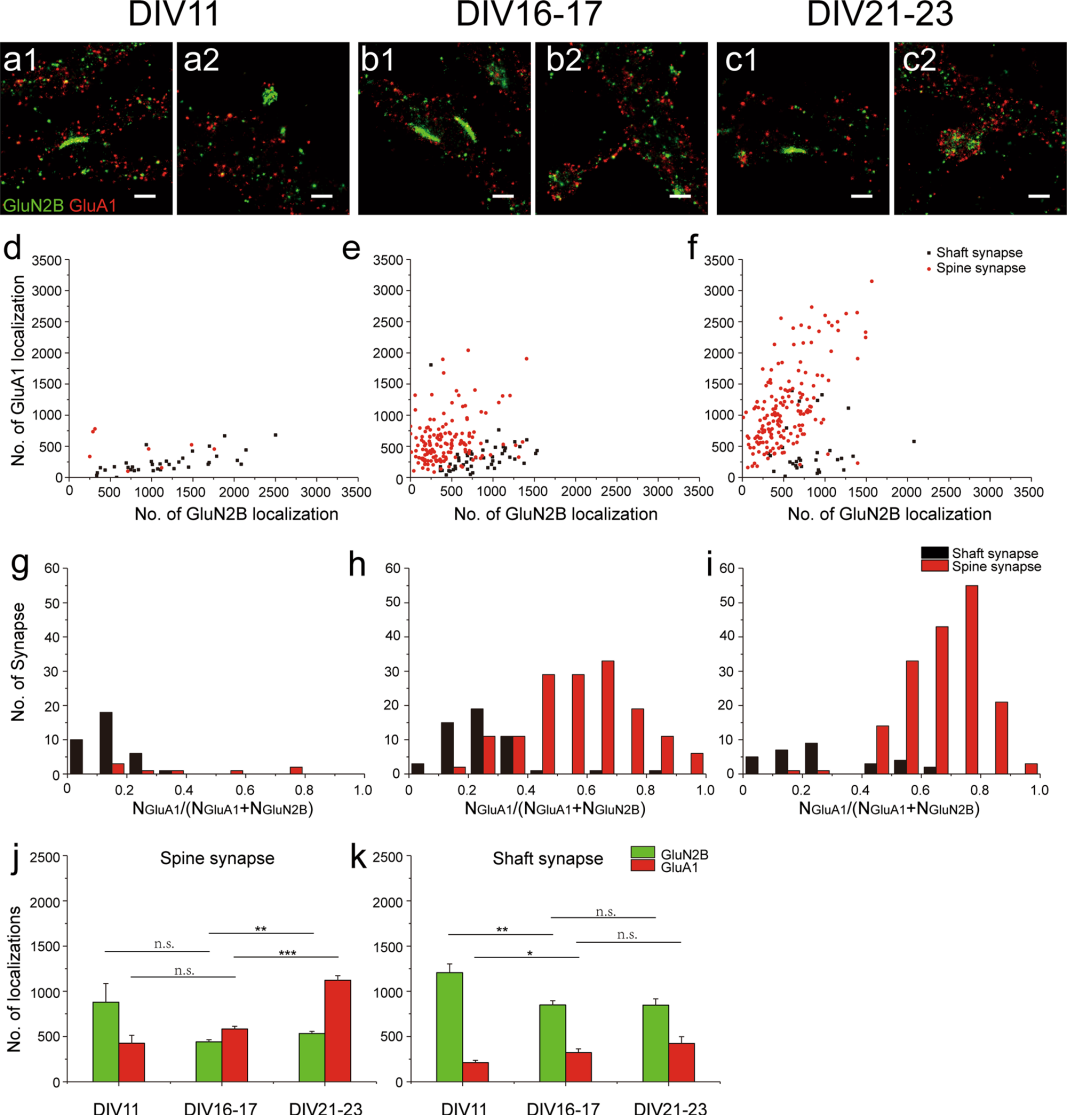
Expression of AMPARs and NMDARs in spine and shaft synapse at different culture stages. **a**–**c** Example dual color STORM images of GluN2B (green) and GluA1 (red) expression in shaft (**a1, b1, c1**) and spine (**a2, b2, c2**) synapses at DIV 11, 16-17 and 21-23. Scale bar, 500 nm. **d**–**f** Scatter plots of GluN2B and GluA1 localizations in shaft (black) and spine (red) synapses in culture stages corresponding to the examples in (**a**–**c)**, respectively. **g**–**i** Histogram of *N*_GluA1_/(*N*_GluA1_ + *N*_GluN2B_) in shaft (black) and spine (red) synapses in culture stages corresponding to the examples in (**a**–**c)**, respectively. **j**–**k** Summary of GluN2B (green) and GluA1 (red) expression in spine synapse (**j**) and shaft synapses (**k**) at different culture stages, with Error bars are standard error of the mean (SEM), with *** denoting *P* < 0.001, ** denoting *P* < 0.01, * denoting *P* < 0.05, n.s. denoting no significance, *t-*test, in this and subsequent Figs unless otherwise noted. *n* = 35 (shaft), 8 (spine) in DIV11; *n* *=* 51(shaft), 151(spine) in DIV16 *-*17; *n* = 30 (shaft), 171(spine) in DIV21-23.

It is known that synapses become enriched in AMPARs during neuronal development and brain maturation^21,29^. With STORM imaging and analyses, we further evaluated receptor expression in individual synapses at different developmental stages. At DIV11, we found that the majority of synapses were silent shaft synapses, with a few spine synapses being either silent (with low AMPAR proportion similar to the silent shaft synapses) or functional (with higher AMPAR proportion) (Fig. 2a, d, g and Supplementary Fig. S7a). The maturation of the neurons was accompanied by a moderate decrease in the density of shaft synapses and a dramatic increase in the density of spine synapses (Supplementary Fig. S7). At DIV 16 -17 and DIV 21-23, the majority of spine synapses contained both AMPARs and NMDARs receptors and with high AMPAR proportion (Fig. 2b, c, e, f, h, i). In contrast, although a few shaft synapses contained high levels of AMPARs (Fig. 2f, i), the majority of shaft synapses at these stages were still silent, expressing much fewer AMPARs as compared to spine synapses (Fig. 2b, c, e, f, h, i). Further analyses revealed that during this period of development (from DIV16 -17 to DIV 21-23), there was a marked increase in the expression of AMPARs and NMDARs for spine synapses (Fig. 2e, f, j). However, the shaft synapses during the same period exhibited no increase in the level of NMDAR expression (Fig. 2e, f, k). We suspected that the NMDA receptor expression level was related to the physical size of shaft and spine synapses. To evaluate this, we first differentiated synaptic and extrasynaptic NMDAR localizations in visually identified synapses based on local density cluster analysis^30^, and then calculated the longest diagonal of the convex hull formed by the identified cluster of synaptic receptors as a measure of synaptic size (Supplementary Fig. S8a-c). Indeed, whereas spine synapses showed significant growth in size (669 ± 69 nm at DIV11,884 ± 29 nm at DIV 16-17, and 1060 ± 32 nm at DIV 21-23), shaft synapses at different stages of neuronal development had similar size (748 ± 24 nm at DIV 11; 809 ± 29 nm at DIV 16-17; 717 ± 37 nm at DIV 21-23) (Supplementary Fig. S8d). We also did the same measurements for AMPARs in DIV 16-23 spine synapses and found that AMPARs occupied a larger area than NMDARs (Supplementary Fig. S8e). To validate these measurements, we compared the data from STORM imaging with that obtained from cryo-electron tomography (cryoET). The mean size of dendritic spines measured by STORM was indeed similar to that based on cryoET measurements (Supplementary Fig. S9).

At DIV16-17 and DIV 21-23, we also noticed a tendency of increased AMPAR localizations in shaft synapses (Fig. 2e, f, k). Aside from possible contaminations from non-specific staining, this might also be related to the expression of dendritic AMPARs during development^21^. When we counted AMPAR localizations in non-synaptic dendritic areas, substantial receptor expression was found at all developmental stages (432.1 ± 31.7, 530.3 ± 18.5, 661.7 ± 26.7 per µm^2^ at DIV11, DIV16-17 and DIV21-23, respectively) (Supplementary Fig. S10). Based on these values, we could estimate the “background” AMPAR localizations for an average shaft synapse (141.0 ± 8.0, 249.8 ± 16.2, 251.1 ± 23.4 AMPAR localizations in shaft synapse at DIV11, DIV16-17 and DIV21-23 respectively). Such “background” could account for a substantial portion of the observed AMPAR localizations in these shaft synapses.

**Fig. 3:**
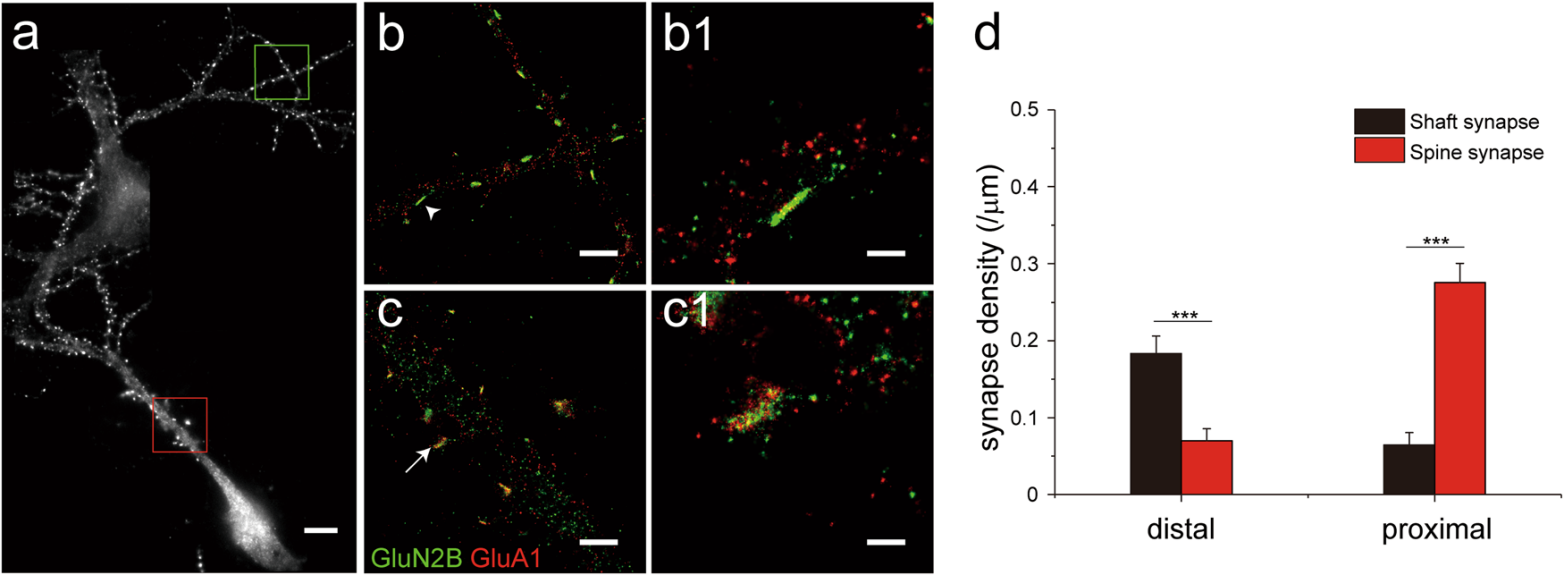
Differential distribution of shaft and spine synapses along neuronal dendrites. **a** Stitched conventional fluorescence images showing distal (green box) and proximal (red box) dendritic segments of a hippocampal neuron. **b** STORM images of the distal segments in green box of (**a**). A magnified view of a shaft synapse, arrow head in (**b**) is shown in (**b1**). **c** STORM images of the proximal segments in red box of (**b**). A magnified view of a spine synapse, arrow in (**c**) is shown in (**c1**). Scale bars in (**a**): 10 µm; (**b**, **c**): 2 µm, (**b1**, **c1**): 500 nm. **d** Summary of synapse density in distal (*n* *=* 21) and proximal (*n* *=* 12) segments for shaft and spine synapses.

In the above analyses, only synapses on proximal dendrites (<50 μm from soma) were included. When synapses on the distal segments (>100 μm from soma) of dendrites in DIV 16-23 cultures were examined, we found that the majority (71.1%) of them were shaft synapses (Fig. 3a, b, b1, d). In contrast, spine synapses were dominant (83.5%) in proximal dendrites of the same neurons (Fig. 3a, c, c1, d). This is consistent with the observation that shaft synapses form earlier in development than spine synapses as distal dendrites are relatively young compared to the proximal segments. Taken together, these results also suggest that the silent shaft synapse could represent a “young” synaptic state, and over time, may be converted into or replaced by functional spine synapses.

**Fig. 4:**
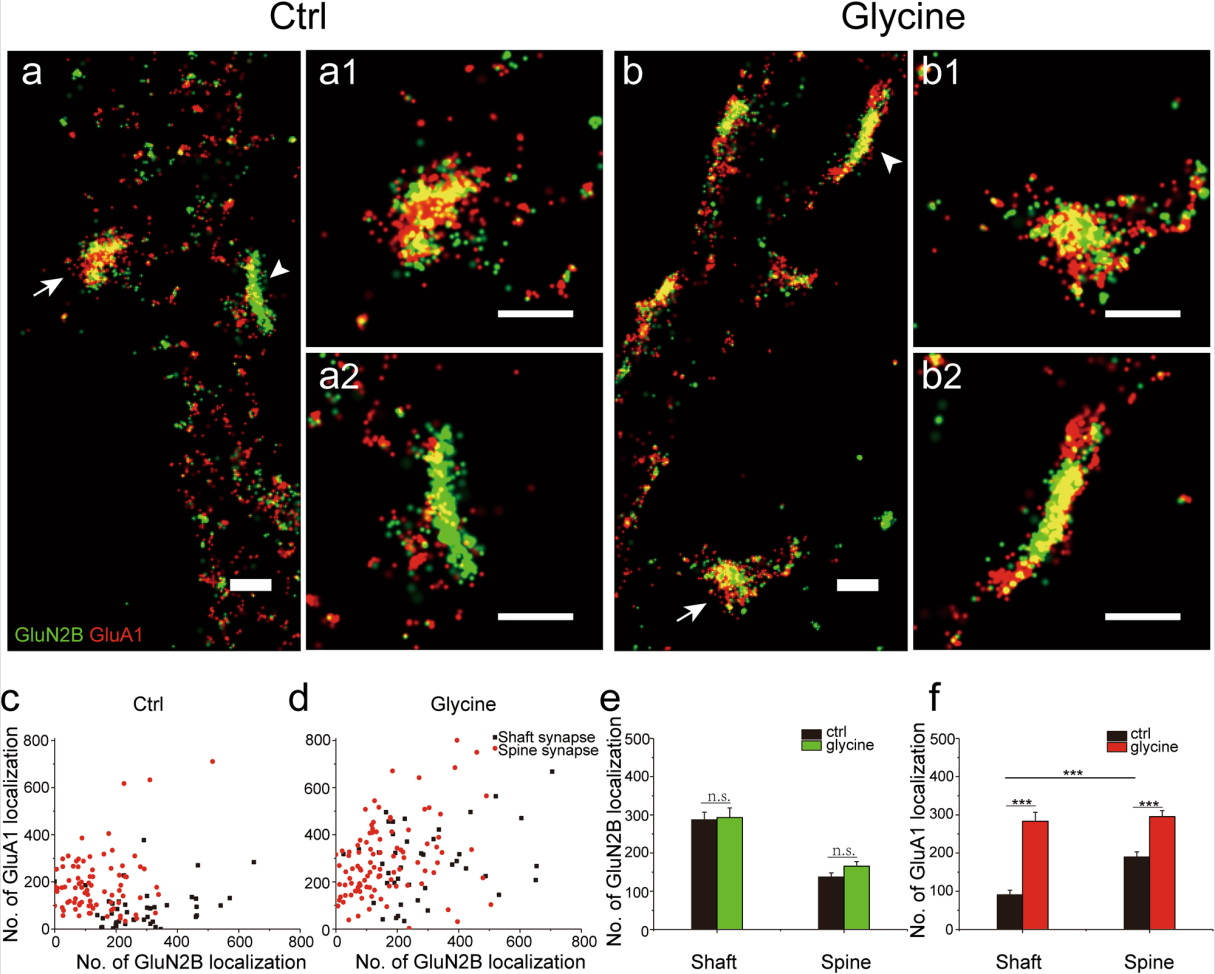
Differential changes of receptor expression in spine and shaft synapse accompanying chemically induced long-term potentiation. **a, b** Dual color STORM images of GluN2B (green) and GluA1 (red) expression in control (**a**) and glycine-stimulated (**b**) neurons, with magnified views of a spine synapse (arrow) shown in **a1** and **b1**, and a shaft synapse (arrow head) shown in (**a2**, **b2**). Scale bars: 500 nm. **c, d** Scatter plots of GluN2B and GluA1 localizations in shaft (black) and spine (red) synapses in control (**c**) and glycine-stimulated (**d**) groups. **e**, **f** Summary of GluN2B (**e**) and GluA1 (**f**) localizations in control and glycine-stimulated groups. *n* = 44 (shaft) and 84 (spine) in control group, and *n* *=* 42 (shaft) and 104 (spine) in glycine-stimulated group.

It is well known that silent synapses characterized by physiological criteria can be rapidly converted into functional ones via activity-dependent synaptic plasticity, e.g. long-term potentiation (LTP)^13,15^. To investigate plasticity-related changes of molecular organization in silent shaft synapses, we used brief glycine exposure to induce chemical LTP (cLTP)^16,31^ in cultured neurons at DIV17-18. In previous studies, we have used the cLTP protocol in the same culture to induce functional changes as measured by patch-clamp recording^32^. Live-cell confocal imaging also revealed long-lasting glycine-induced spine enlargement, confirming the effectiveness of the protocol (Supplementary Fig. S11). With STORM imaging, we observed dramatic recruitment of AMPARs within the postsynaptic area of shaft synapses in cLTP group (Fig. 4), as well as overall increases in synaptic size (Supplementary Fig. S12a, b, e). Quantitative analysis (see Methods)^28^ revealed that whereas cLTP did not significantly alter the synaptic content of NMDARs for either shaft or spine synapse (Fig. 4e), it caused substantial increase in the synaptic content of AMPARs for both synapse types (Fig. 4f). Furthermore, cLTP apparently recruited more AMPARs to shaft synapses (AMPAR localizations from 90.5 ± 12.2 in control group to 283.5 ± 23.3 in glycine-stimulated group) than to spine synapses (from 189.6 ± 13.5 to 295.1 ± 16.0), such that the resulted AMPARs in the two types of synapses reached a similar level (Fig. 4f). There was no substantial change in the proportion of spine synapses during cLTP (65.6%, 84 out of 128 in control group and 71.2%,104 out of 146 in glycine-stimulated group). Therefore, the silent shaft synapses were more “potentiable” than spine synapses. Notably, AMPARs in the “potentiated” shaft synapses (most of which were presumably silent prior to the cLTP induction) generally occupied longer distribution length than NMDARs did (Fig. 4b and Supplementary Fig. S13), similar to functional spine synapses (Fig. 2b, c and Supplementary Fig. S8e) and consistent with the observation that LTP involved extrasynaptic insertion and lateral diffusion of AMPARs^33,34^.

## Discussion

The high resolution and molecular specificity offered by STORM imaging allow for visualization of various structural features of excitatory synapses in developing hippocampal neurons, and raise interesting issues regarding their functionality. In particular, we observed that a large number of synapses were formed directly on dendrite shafts and that the majority of silent synapses were shaft synapses, lacking postsynaptic compartmentalization imposed by the thin neck of dendritic spines^35,36^. Thus, compared to the functional spine synapses, the silent shaft synapses would have spatially less confined signaling, such as previously observed spread of calcium^14^, during plasticity induction. This property may also allow for easier recruitment of external resources for plasticity expression. Indeed, we observed a greater increase in AMPARs content in shaft synapses than in spine synapses after cLTP induction, consistent with recent observations that adding AMPARs to established functional synapses required more remodeling events than to synapses with few receptors^37^ (Fig. 4). A possible mechanism is the lack of a restricting spine neck that may allow for easier recruitment of additional molecules to shaft synapses. Furthermore, we observed that after cLTP induction, the localization number of AMPARs in a shaft synapse was similar to that in a spine synapse, suggesting that both types of synapses may have similar number of “slots” for AMPARs^38,39^.

We observed more silent shaft synapses in early developmental stages, suggesting that they may represent a form of “young” synapses that eventually “maturate” into functional spine synapses, as has been noted in various systems^9^. Apparently, this maturation process can be accelerated by LTP induction, with fast formation of functional shaft synapse (that expresses AMPARs) as an intermediate stage. Similar functional shaft synapses were indeed observed in cultures of different stages (Fig. 2e, f). Thus, with super-resolution fluorescence imaging, we have identified at least four structurally distinct classes of excitatory synapses: the silent shaft synapses, the functional shaft synapses, the silent spine synapses, and the functional spine synapses. Among them, the functional shaft synapses and silent spine synapses were less frequent, suggesting that they may represent “transitional” states during synaptic development and plasticity. Such structural heterogeneity is likely to underlie different functional states of synapses possessing distinct properties in synaptic plasticity^7,8,40^. This could permit richer dynamics in the modification of neural circuits, and thus play important roles in learning and memory functions as suggested by theoretical studies^41^. Furthermore, it is possible that more synaptic states can be revealed when the spatial expression of additional synaptic proteins are evaluated. Along this line, future studies may reveal the functional correlates of these synaptic states and the transition among them.

## Materials and methods

### Cell culture

Primary culture of hippocampal neurons were prepared following established protocol^32^ with minor modifications. Briefly, hippocampi were dissected out from brains of fetal rat at embryonic day 18, followed by digestion in 0.25% trypsin (Sigma, St. Louis, MO, USA) for 15 mins at 37°C. The digested tissues were then washed twice with Hank’s Balance Salt Solution (HBSS) buffer (Invitrogen San Diego, CA, USA) and triturated with a fire-polished glass pipette in plating medium (containing neurobasal medium (Invitrogen) supplemented with 1% Glutamax (Invitrogen), 2% B27 (Invitrogen), 1% 3.75 M NaCl (Sigma), 0.1% 25 mM l-glutamic acid (Sigma)). Cells were plated at densities of 30–40/mm^2^ on poly-l-lysine (Sigma) coated glass coverslips (Assistant, Sondheim, Germany) in petri dishes (Corning, Oneonta, NY, USA), and then grown in incubators maintained at 37°C and 5% CO_2_. At DIV 5, half of the culture medium was replaced with maintenance medium which is similar to the plating medium without addition of 0.1% 25 mM l-glutamic acid. Afterwards, 20% of the culture medium was replaced with fresh maintenance medium every 3 – 4 days. For cryoET imaging, primary culture of hippocampal neurons were grown on poly-l-lysine coated gold EM grids (Quantifoil, Au NH2 R2/2) as described previously^20,42^.

### Chemical LTP induction

For cLTP induction, neurons grown on coverslips were first transferred to Mg^2+^-free extracellular solution (containing 150 mM NaCl, 3 mM KCl, 3 mM CaCl_2_, 10 mM HEPES, 5 mM glucose, 0.5 μM tetrodotoxin, 1 μM strychnine, 20 μM bicuculline methiodide, all from Sigma) and were incubated at room temperature for 10 mins. Stimulation was given by 3-min exposure to 200 μM glycine (Sigma) in the same Mg^2+^-free extracellular solution. After glycine stimulation, the coverslips were transferred back to original Mg^2+^-free extracellular solution for 20 min, followed by immunofluorescence staining and imaging.

### Antibody labeling and immunostaining

Cyanine Dye3 (3 μg, GE Healthcare, Little Chalfont, Buckinghamshire, UK) or AlexaFluor405 (3 μg, Invitrogen, Eugene, Oregon, USA) was mixed with 1 μg AlexaFluor647 (Invitrogen), 10 μmol NaHCO_3_ and 100 μg antibody (Jackson ImmunoResearch, West Grove, PA, USA) in 100 μl PBS with gentle agitation at room temperature for 30 min. During reaction the Nap5 gel-filtration column (GE Healthcare) was equilibrated with 3 volumes of PBS. A UV–Vis spectrophotometer was used to detect the the number of dye labeled in single antibody (1.5–3.0 activator and 0.4-0.8 reporter labeled in one antibody should be perfect for multi-color STORM imaging). Cultured neurons were fixed by 20-min incubation in PBS (137 mM NaCl, 2.7 mM KCl, 10 mM Na_2_HPO_4_, 2 mM KH_2_PO_4_) containing 3% paraformaldehyde, then permeabilized by 0.2% Triton-X100 in PBS for 6 mins. After 1-h blocking with 3% BSA (in PBS), the sample were incubated in 3% BSA (in PBS) containing appropriate one or more of the following primary antibodies: rabbit-anti-GluA1 (31232 from Abcam, Cambridge, MA, USA), mouse-anti-Bassoon (13249 from Abcam), mouse-anti-GluN2B (610416 from BD Bioscience, San Jose, CA, USA), or guinea pig-anti-vGlut (135304 from Synaptic system, Gottingen, Germany) at 4°C overnight (20 μg/ml GluA1 and 5 μg/ml GluN2B for STORM staining), followed by incubation in appropriate fluorescently labeled secondary antibodies (Rabbit antibody conjugated with Cy3-Alexa647 STORM pair labeled to GluA1, and mouse antibody conjugated with Alexa405-Alexa647 STORM pair labeled to GluN2B or bassoon, Guinea pig conjugated with Alexa488 binding to vGlut when necessary)(Jackson ImmunoResearch) at room temperature for 40 mins. Post fixation for 20 min with 3% paraformaldehyde in PBS was performed to preserve fluorescent signals for longer periods of storage. All antibody dilution ratio and incubation time were kept consistent in control and glycine stimulation group.

### STORM imaging

All imaging experiments were performed on a custom built STORM setup. The optical system consists of an inverted fluorescence microscope (Olympus, Tokyo, Japan) with a 100X oil NA1.4 objective, a translational stage (Applied Scientific Instrumentation, Eugene, OR, USA), a set of solid state lasers with output wavelengths of 405, 460, 488, 639 nm (Coherent, Santa Clara, CA, USA), 560 nm (MPB, Pointe-Claire, QC, Canada) and 532 nm (Oxxius) to provide controlled illumination light through an AOTF (Crystal Technology Inc., Palo Alto, CA, USA), an EMCCD (Andor, Belfast, UK) attached to the microscope through a Dual View image splitter (Photometrics, Tucson, AZ, Canada), into which a cylindrical lens of 1 m focal length were inserted for 3D STORM.

Before STORM imaging, fixed cells were immersed in fresh imaging buffer containing 80% PBS, 10% 50%(w/v) Glucose, 10% 1 M mercaptoethylamine, with addition of 1% oxygen scavenger buffer made by 8 mg glucose oxidase and 160 μg catalase dissolved in 100 μl PBS after sufficient mixing and 1 min centrifuge. Weak 639 nm illumination was used to acquire conventional wide-field images of the samples and to identify areas of interest containing healthy dendritic and synaptic structures, usually within 50μm from the soma for proximal synapses or at least 100 μm from the soma for distal synapses. In subsequent STORM image acquisition, time series of single molecule fluorescence images were acquired at 60 Hz, with a periodic illumination pattern consisting of one activation frame followed by three imaging frames^28^. Averaging all frames of single molecule signals also results in equivalent “wide-field” images.

For STORM imaging in cLTP experiments, activation-imaging cycles were repeated until virtually all fluorophores in both GluN2B and GluA1 channels depleted. This together with consistent fluorophore labeling and antibody staining allowed for fair comparison of receptor levels in shaft and spine synapses in control and glycine groups.

### STORM data processing

Identification and fitting of single molecule localizations, as well as STORM image reconstruction were conducted using custom software as previously described^28^. In STORM imaging, one “localization” refers to an on-off switching (blinking) event of a single fluorescence molecule (Alexa647) captured by the high-speed camera. Typically, a receptor protein was labeled by a few secondary antibody molecules, and each Alexa647 fluorophore on an antibody molecule could blink multiple times before being photo-bleached^24^. Thus, the measured number of localizations should be largely proportional to, but generally much larger than the actual number of antigen (e.g. AMPAR or NMDAR) protein molecules. We used the localization number of each identified synapse to quantify the relative expression level of synaptic proteins, as did in previous studies^28^.

Synapses along selected dendritic segments in reconstructed STORM images were visually identified based on the morphological features revealed by localizations of both dendritic AMPARs and synaptic proteins. The relatively even distribution of dendritic GluA1 localizations allowed for visualization of dendritic shaft profiles (Supplementary Fig. S3), whereas the clustered distribution of bassoon, AMPAR and NMDAR localizations helped identify pre- and postsynaptic compartments (Figs. 1b–d, 2a–c, 3b, c, 4a, b); Supplementary Fig. S3). At STORM resolution, spine synapses, even those with very short necks could be identified rather easily because the position of their synaptic protein clusters were away from dendritic shaft profiles (Fig. 1b1 and Supplementary Fig. S1d-f). Meanwhile, shaft synapses could be identified based on their distinct line-shaped clusters of GluN2B localizations that were located inside the dendritic shaft profiles (Fig. 1b2 and Supplementary Fig. S1d-f). About 20% of receptor clusters could not be identified as spine or shaft synapses based on the above criteria and were categorized as “uncertain” type.

Synaptic expression of AMPARs and NMDARs were quantified by counting the total localization numbers within ROIs that enclosed identified postsynaptic compartments. Dendritic AMPAR expression density was evaluated based on the number of AMPAR localizations within randomly selected dendritic areas. The average AMPAR expression density was then multiplied by the area of an identified shaft synapse (calculated from *x*, *y* coordinate in reconstructed image) to obtain the “background” expression level of AMPARs. At the first order approximation, this localization number is proportional to the number of target protein molecules for the same batch of experiments when the labeling and imaging conditions are kept the same.

### Calibration of crosstalk for dual color STORM imaging

We noticed that signals from the Cy3-Alexa647 channel (GluA1) could be detected in presynaptic areas and account for ~7% of total localizations from both channels, whereas ~15% localizations in the postsynaptic area were from the Alexa405-Alexa647 channel (bassoon). This could come from non-specific antibody binding and fluorescence activation crosstalk between the two channels (i.e. Alexa405-Alexa647 pair vs Cy3-Alexa647 pair). The latter was minimized by the following calibration procedure.

Assuming that the acquired localization numbers from the two channels are *D*_1_ and *D*_2_, which are from the real signals *d*_1_ and *d*_2_. Considering non-specific activation *b*_1_ (the portion of *d*_1_ measured as *D*_2_) and *b*_2_ (the portion of *d*_2_ measured as *D*_1_), a set of transfer equations can be expressed as

1 $${{D}}_1 = {{a}}_1 \ast {{d}}_1 + {{b}}_2 \ast {{d}}_2,$$

2 $${{D}}_2 = {{b}}_1 \ast {{d}}_1 + {{a}}_2 \ast {{d}}_2,$$

3 $${{a}}_1 + {{b}}_1 = 1,$$

4 $${{a}}_2 + {{b}}_2 = 1.$$

The parameters *a*_1_, *a*_2_, *b*_1_, *b*_2_ can be obtained from two calibration experiments using samples containing single fluorophores (i.e., *d*_1_=0, *D*_1_ + *D*_2_ = *d*_2_ for one condition, and *d*_2_ = 0, *D*_1_ + *D*_2_ = *d*_1_ for the other).

Solving Eqs. (1) and (2), the calibrated localization number *d*_1_ and *d*_2_ can be expressed as

$${{d}}_1 = {\upalpha}_1 \ast {{D}}_1 + {\upbeta}_2 \ast {{D}}_2,$$

$${{d}}_2 = {\upbeta}_1 \ast {{D}}_1 + {\upalpha}_2 \ast {{D}}_2.$$

The parameters ɑ_1_, β_1,_ ɑ_2_, β_2_ are

$${\upalpha}_1 = {{a}}_2/({{a}}_1 \ast {{a}}_2 - {{b}}_1 \ast {{b}}_2),$$

$${\upbeta}_1 = {{b}}_1/({{b}}_1 \ast {{b}}_2 - {{a}}_1 \ast {{a}}_2),$$

$${\upalpha}_2 = {{a}}_1/({{a}}_1 \ast {{a}}_2 - {{b}}_1 \ast {{b}}_2),$$

$${\upbeta}_2 = {{b}}_2/({\mathrm{b}}_1 \ast {{b}}_2 - {{a}}_1 \ast {{a}}_2).$$

In this study, the calibrated localizations were used for all quantitative analysis of NMDAR and AMPAR expression in shaft and spine synapses. In a typical experiment, for example, where *D*_1_ and *D*_2_ were measured as the numbers of localizations for Alexa405-Alexa647 and Cy3-Alexa647 channels, respectively. We obtained that *a*_1_=0.954, *a*_2_=0.842, *b*_1_=0.046, *b*_2_=0.158, thus *ɑ*_1_ = 1.0578, β_1_ = -0.0578, *ɑ*_2_ = 1.1986, β_2_ = −0.1986_._

### Identification of silent synapses

For identified excitatory synapses in Fig. 1e, the AMPAR proportion values defined as *N*_GluA1_/(*N*_GluA1_ + *N*_GluN2B_) exhibited bimodal characteristics, and was well fitted with two Gaussians (Supplementary Fig. 5), one representing silent synapse population and the other functional synapse population. The intersection of the two curves was at *N*_GluA1_/(*N*_GluA1_ + *N*_GluN2B_) = 0.37. Empirically, we used this value to separate silent synapses from functional synapses. A synapse with low AMPAR proportion, i.e. *N*_GluA1_/(*N*_GluA1_ + *N*_GluN2B_) < 0.37, is considered a silent synapse.

### Differentiation of synaptic and extrasynaptic localizations

We adapted a local density analysis approach similar to that reported in previous studies^30^ to distinguish synaptic localization signal and background noise. In brief, randomly distributed *N* (*n* = 8000–400,000) localizations in an area of *S* = 1600μm^2^ were simulated with an average density *d* = *N*/*S*. For each localization, its nearest neighbor distance was obtained as NND(i). Then the median NND (mNND) of all N points for each simulation was obtained. Through fitting a series of mNNDs versus average densities, a standard median NND (stmNND) was calculated as a function of the average density, which equals 471/sqrt(d).

For one specific STORM image of neuron, the average density of whole dendritic region and its specific stmNND were calculated first based on simulated stmNND function. The local density corresponding to each localization was defined as the number of neighboring localizations within 2.5 times stmNND. Then, localizations in visually identified synapse with higher local density than the average of all localizations in the dendritic region was considered as signal and the rest as background noise (Supplementary Fig. S8a2, b2, c2).

After all of STORM signal was identified by local density, the longest diagonal of the convex hull of all receptor localizations within the synapse was defined as a measurement of synaptic length (Supplementary Fig. S8a3, b3, c3).

### Live cell imaging of glycine stimulation

We transfected plasmid of actin-mcherry in DIV11. After 6-7days expression, Cells were transferred to a custom chamber for live cell imaging. during imaging acquisition cells were perfused in more than 10 mins Mg^2+^-free extracellular solution as baseline, and 3 mins 200 μM glycine in the same Mg^2+^-free extracellular solution, and more than 20 mins Mg^2+^-free extracellular solution in sequence. During the whole procedure, N.A.1.45 oil objective in spinning disk confocal microscopy was used to monitor synapse morphology change.

### CryoET imaging

Neuronal cultures on EM grids in DIV16 were transferred to extracellular solution (ECS containing 150 mM NaCl, 3 mM KCl, 3 mM CaCl2, 2 mM MgCl2, 10 mM HEPES, and 5 mM glucose, pH 7.3), and then rapid vitrified with a plunge freezer (Vitrobot IV, FEI, Netherland). The frozen grids were stored in liquid nitrogen until use.

CryoET data was collected using a 200KV transmission electron microscope (Tecnai F20, FEI) equipped with a K2 Summit direct electron detector (K2 camera, Gatan). Tilt series were collected from 0° to −54°, and then from + 3° to + 60°, at of 3° intervals using SerialEM^43^, with the defocus value set at −6 to −10 µm, and the total electron dosage of 100 e/Å^2^. The images were acquired using K2 camera in counting mode with a final pixel size of 0.565 nm. Tilt series were aligned and reconstructed using IMOD^44^. The measurement of PSD was performed using 3dmod in IMOD package.

### Statistical information

Statistics were presented as Mean ± SEM, with ***P *<* 0.001, **P *<* 0.01, *P *<* 0.05, n.s. denoting no significance. Two tailed *t*-test was used to verify statistical difference between two groups. Paired *t*-test was used to determine statistical difference between GluN2B and GluA1 distribution length within synapses. For all statistical tests, *P* value < 0.05 was considered as statistical significant difference.

All supporting data are available from the authors upon request.

## Supplementary information

Supplementary Information

Supplementary movie S1

Supplementary movie S2

## Supplementary information

**Supplementary Information** accompanies the paper at (10.1038/s41421-019-0139-1).
