# Supplementary material for: Structure and plasticity of silent synapses in developing hippocampal neurons visualized by super-resolution imaging
Source: Cell Discov. 2020 Feb 25;6:8. doi: 10.1038/s41421-019-0139-1 (PMC7039918; doi:10.1038/s41421-019-0139-1)
Supplement: Supplementary file 1 — Supplementary Information [file 41421_2019_139_MOESM1_ESM.docx]

**Supplementary Information**

**Supplementary figures**

**Supplementary Fig. S1**


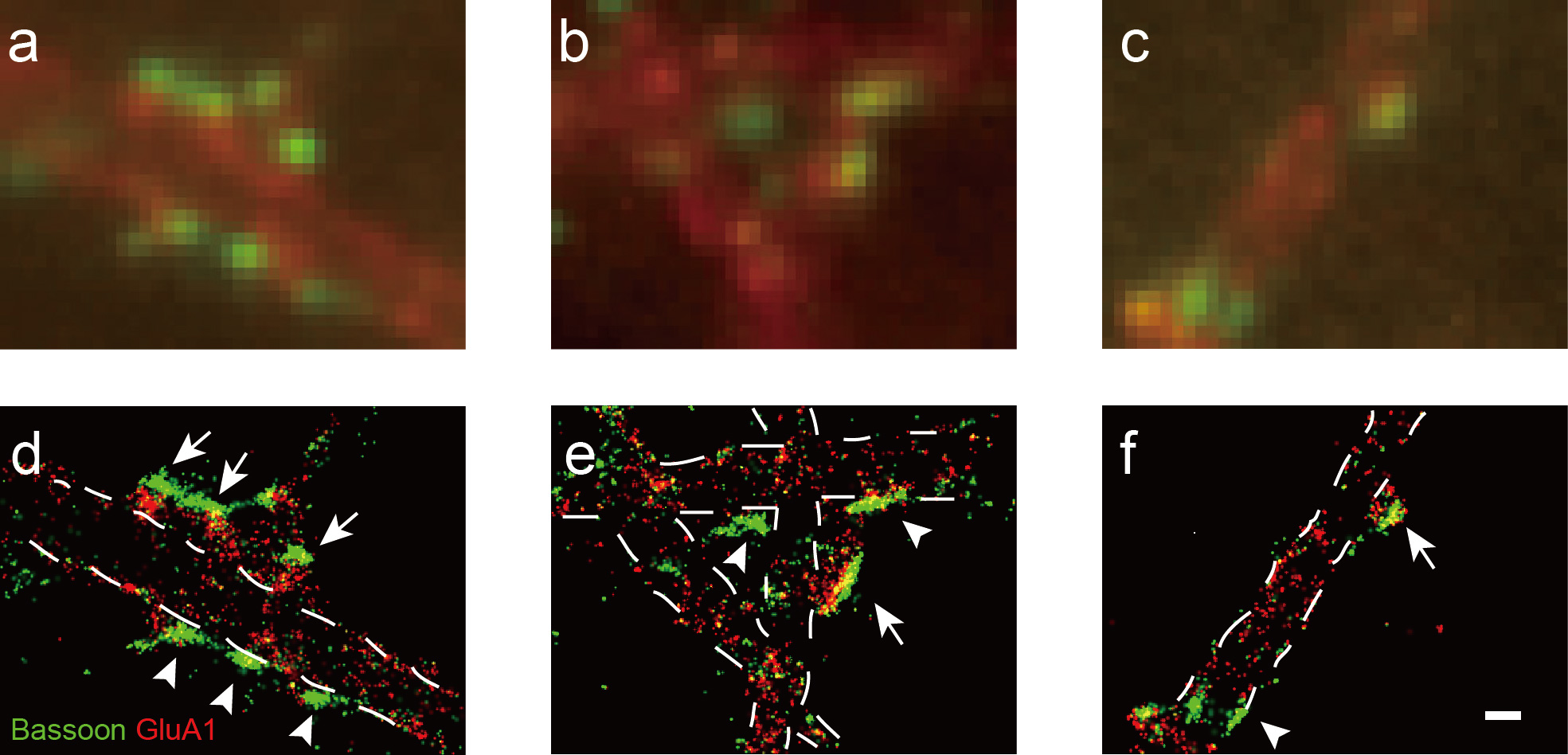


**Supplementary Fig. S1**. Examples of spine and shaft synapses under conventional and STORM imaging.

With fluorescent signals of presynaptic scaffolding protein bassoon (green) and postsynaptic receptor GluA1 (red), spine synapses (arrow) and shaft synapses (arrow head) synapses difficult to identify in conventional imaging (**a, b, c**) could be distinguished with STORM (**d, e, f**). Dashed lines were drawn based on the distribution of dendritic GluA1 localizations to show dendritic shaft profiles. Scale bar: 500nm.

**Supplementary Fig. S2**


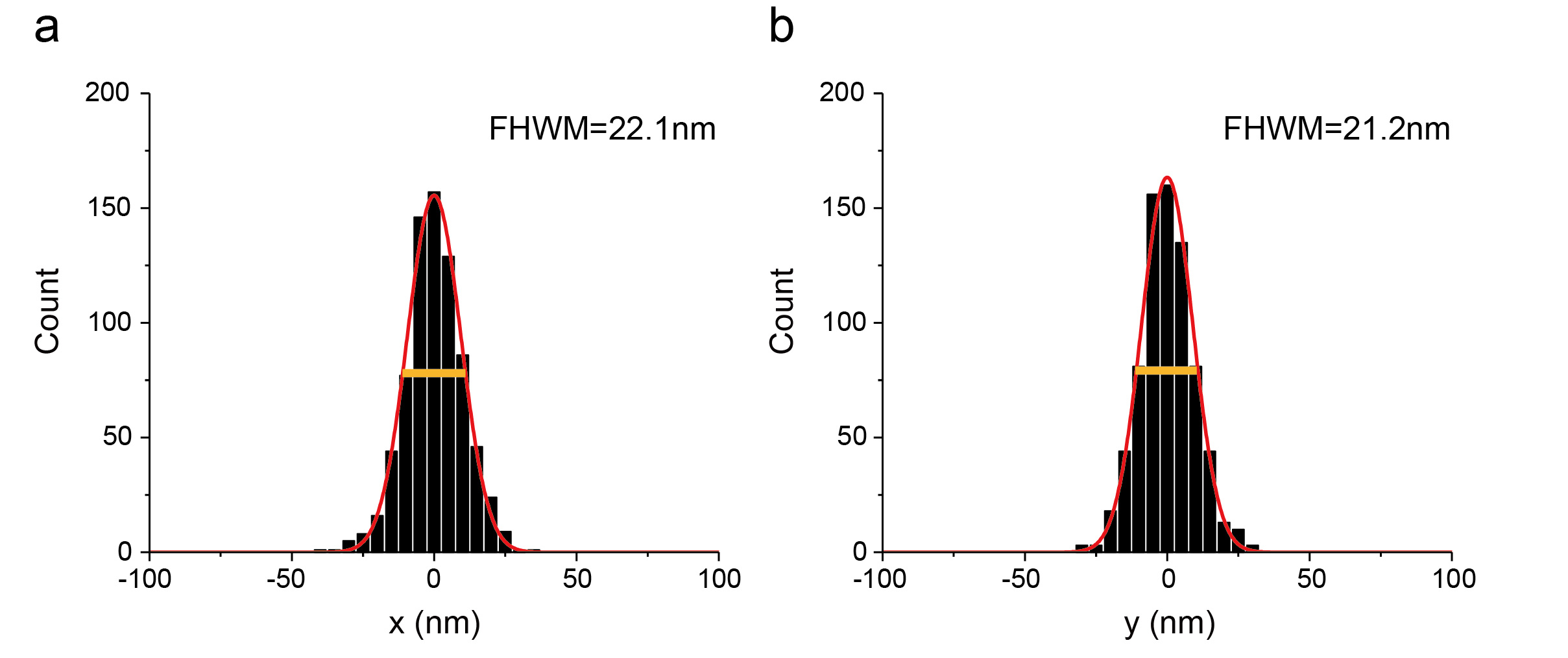


**Supplementary Fig. S2.** Characterization of STORM resolution.

Data from localization clusters of 59 fluorophores in sparsely labelled samples were collected to obtain a summation of localization count distribution in x (**a**) and y (**b**) dimensions. Gaussian fitting of the histogram (red curve) yields Full Width of Half Maximum (FWHM, yellow line) of 22nm (x) and 21nm (y) which is considered as the effective resolution of the system.

**Supplementary Fig. S3**


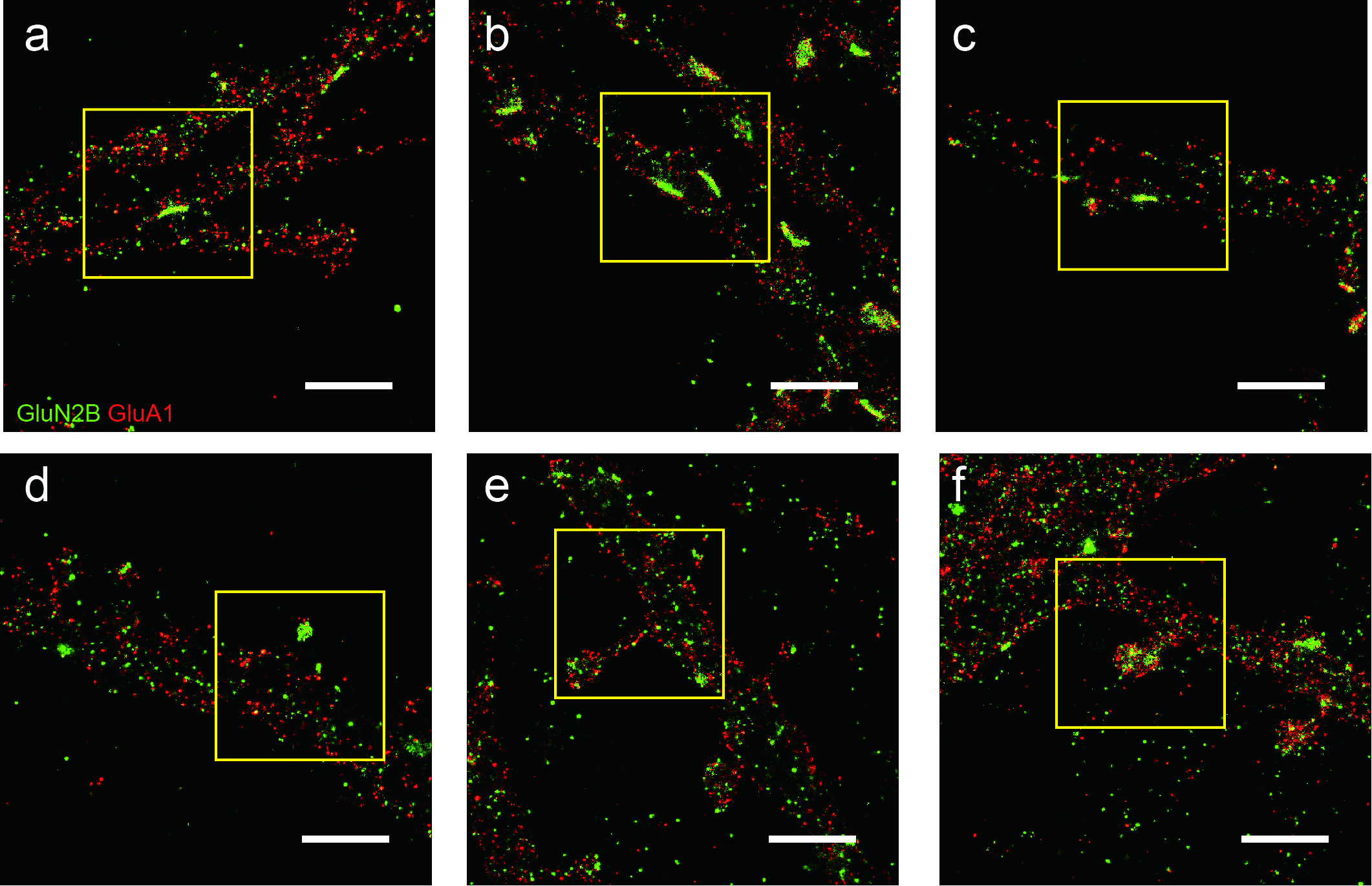


**Supplementary Fig. S3**. Low-zoom STORM images to show dendritic profiles.

At this zoom level, dendritic profiles are easily visualized based on STORM localizations of dendritic GluA1 at high contrast**.** Distinct line-shaped clusters of GluN2B (green) localizations on the dendrite are identified as shaft synapses **(a-c)**. Other GluN2B (green) and GluA1 (red) localizations away from the dendrites are identified as spine synapses **(d-f)**. Yellow squares indicate the area of synapses shown in **(Fig. 2)**. Scale bar: 2µm.

**Supplementary Fig. S4**


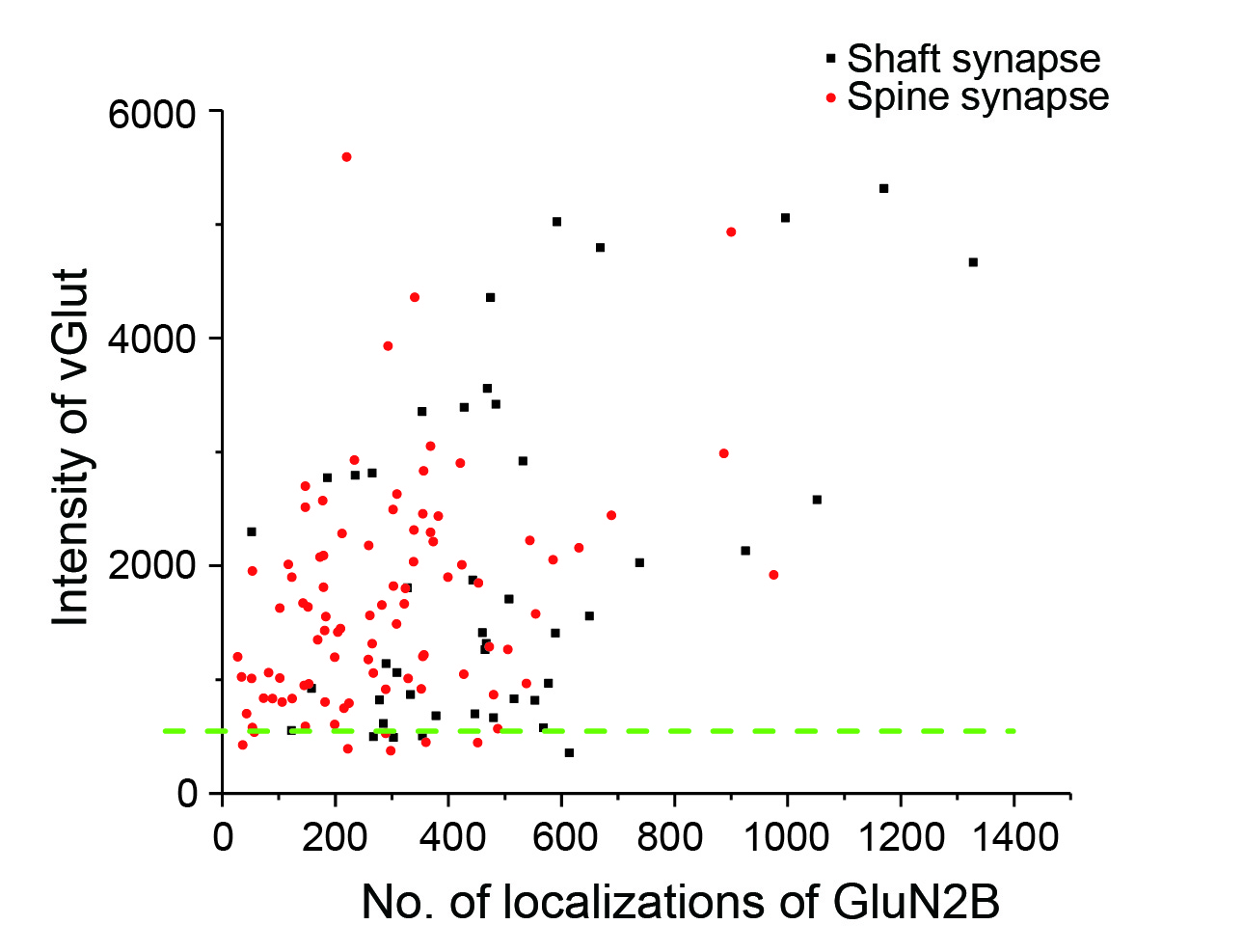


**Supplementary Fig. S4**. Correlation of presynaptic vGlut and postsynaptic GluN2B.

Scatter plot shows the maximum intensity of vGlut signal (in conventional image) vs the number of GluN2B localizations (in STORM image) for putative shaft (black dots) and spine (red dots) synapses. The vast majority of data points are above the background level of vGlut signal in dendrites (green dashed line), indicating that virtually all GluN2B clusters have corresponding vGlut puncta.

**Supplementary Fig. S5**


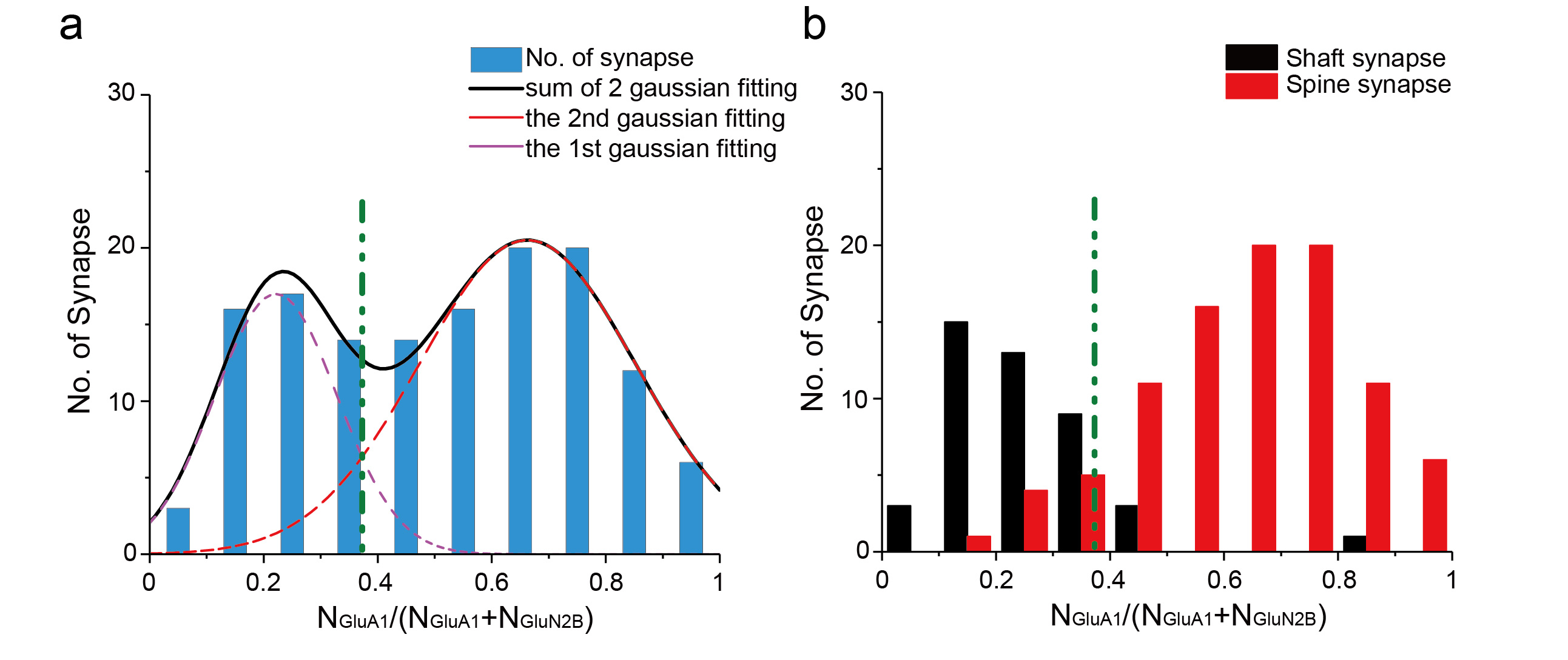


**Supplementary Fig. S5**. Bimodal distribution of AMPAR proportion (defined as N_GluA1_/(N_GluA1_+N_GluN2B_)) of synapses in DIV 17 cultures.

**a** The histogram of N_GluA1_/(N_GluA1_+N_GluN2B_) of all synapses can be fitted as a sum of 2 Gaussians (black curve), with the two components (violet and red dashed curves) corresponding to silent and functional synapse populations. Green line shows the intersection of the 2 component curves at N_GluA1_/(N_GluA1_+N_GluN2B_)=0.37. **b** Histograms of AMPAR proportion for shaft (black) and spine (red) synapses.

**Supplementary Fig. S6**


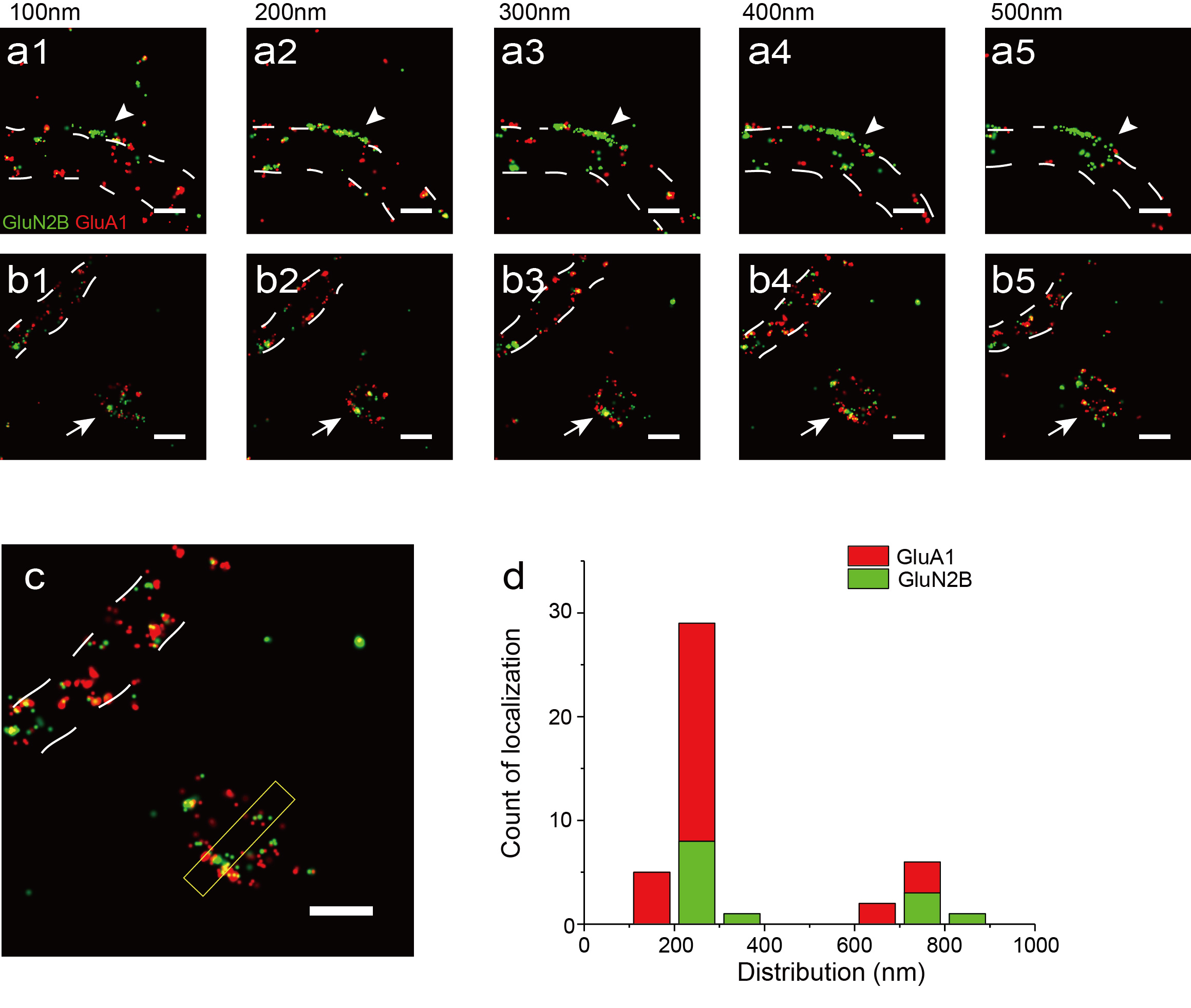


**Supplementary Fig. S6**. 3-D STORM imaging of a silent shaft synapse and a spine synapse.

**a-b** Z-stack slices of GluN2B (green) and GluA1 (red) staining of a shaft synapse (arrow head in **a**) and a spine synapse (arrow in **b**). Dashed lines were drawn based on the distribution of dendritic GluA1 localizations to show dendritic shaft profiles. **c** Magnified view of the same 100-nm thick cross-section of the spine synapse as in (**b4**). **d** Quantification of GluA1 (red) and GluN2B (green) localization counting along the axis of the 200nm-wide bar in (**c**). Scale bar: 500nm.

**Supplementary Fig. S7**


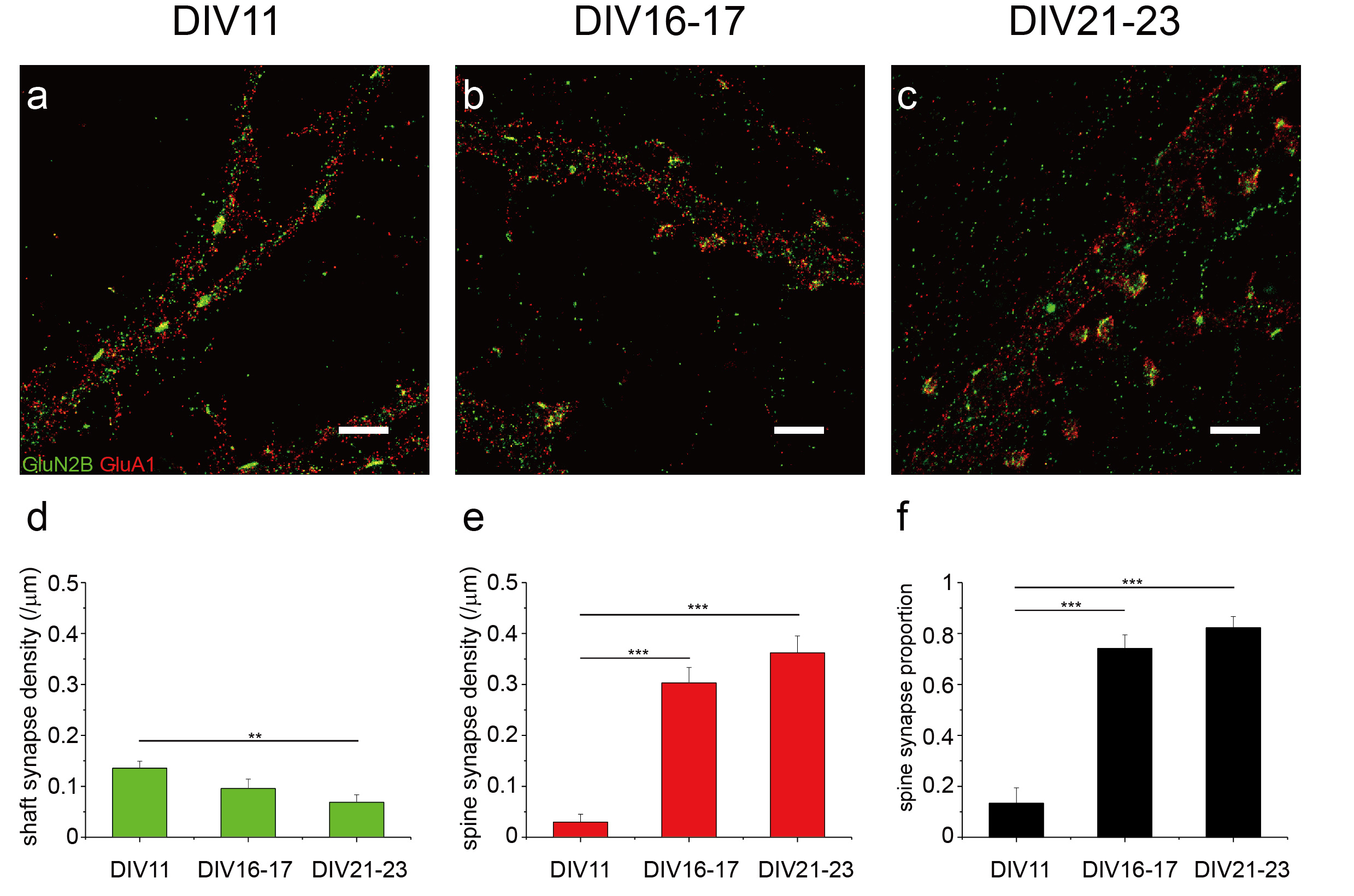


**Supplementary Fig. S7.** Changes in the proportions of spine and shaft synapses during development.

**a-c** STORM images of GluN2B (green) and GluA1 (red) staining in cultured neurons at DIV11 (**a**), DIV16-17 (**b**), DIV21-23 (**c**). Scale bar: 2μm. **d-e** Statistics of dendritic densities of shaft (**d**) and spine (**e**) synapses. N=13 for DIV11, N=16 for DIV16-17, N=18 for DIV21-23. **f** Statistics of proportion of spine synapses over development.

**Supplementary Fig. S8**


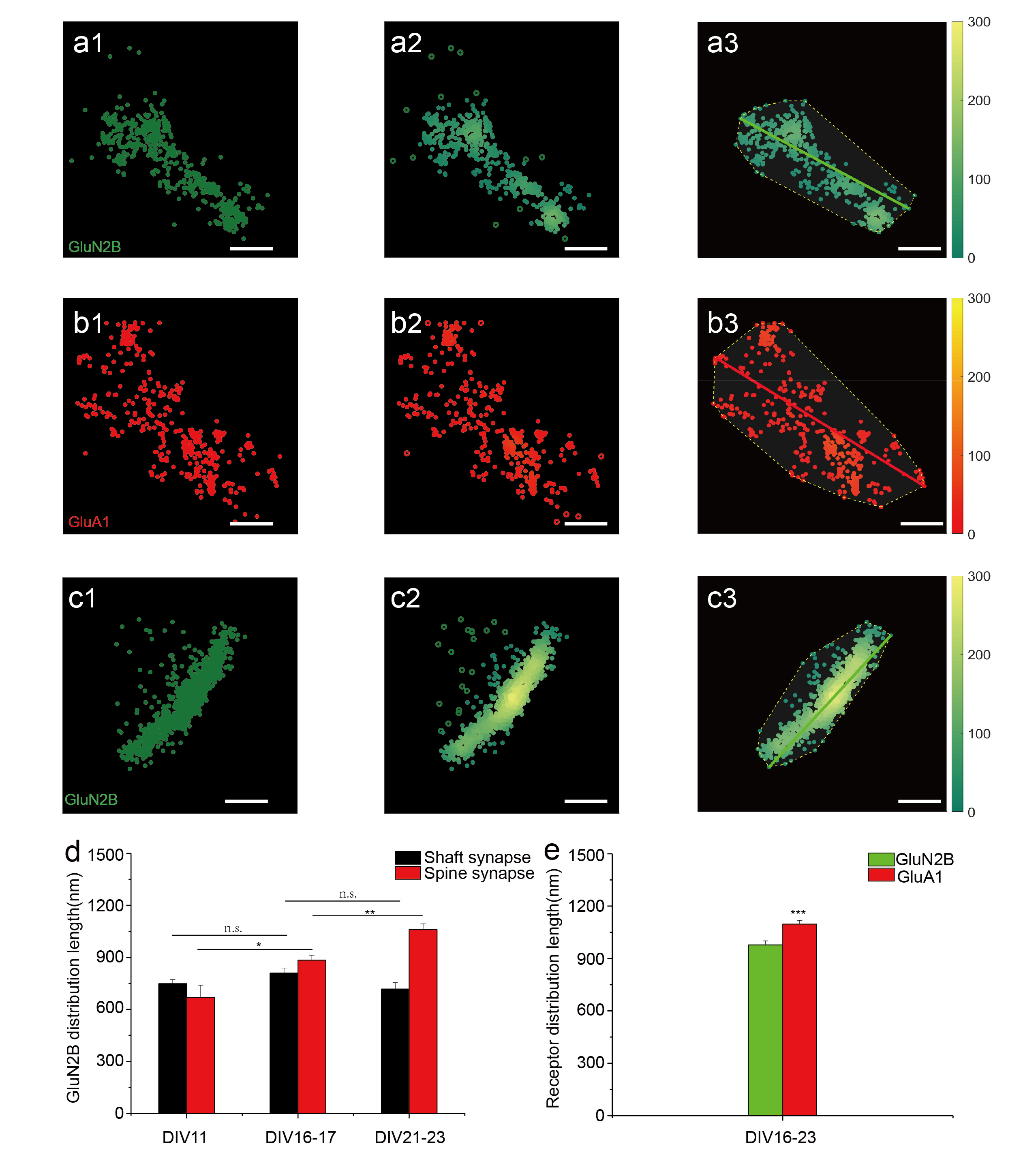


**Supplementary Fig. S8.** Assessment of changes in synaptic size during development.

**a-b** Size measurement of a spine synapse based on spatial distribution of identified synaptic AMPA or NMDA receptor clusters. STORM images of NMDA (**a1**) and AMPA (**b1**) receptor expression in a visually picked synapse were processed by local density analysis (see Appendix) to classify localizations into synaptic (solid dots in **a2** and **b2**) and non-synaptic (hollow dots in **a2** and **b2**) classes. The size of a synapse was measured by the longest diagonal of the convex hull formed by identified synaptic AMPA (**a3**) or NMDA (**b3**) receptors. Scale bar: 200nm. **c** Size measurement of a shaft synapse based on its NMDA receptor expression using the same procedure as in **a**. **d** Comparison of synaptic sizes at different developmental stages based on the distribution of GluN2B receptors in shaft (black) and spine (red) synapses. N=35(shaft), 8(spine) for DIV 11; N=51(shaft), 151(spine) for DIV 16-17; N=30(shaft), 171(spine) for DIV 21-23. **e** Comparison of distribution length of GluN2B (green) and GluA1 (red) in spine synapses. N=322.

**Supplementary Fig. S9**


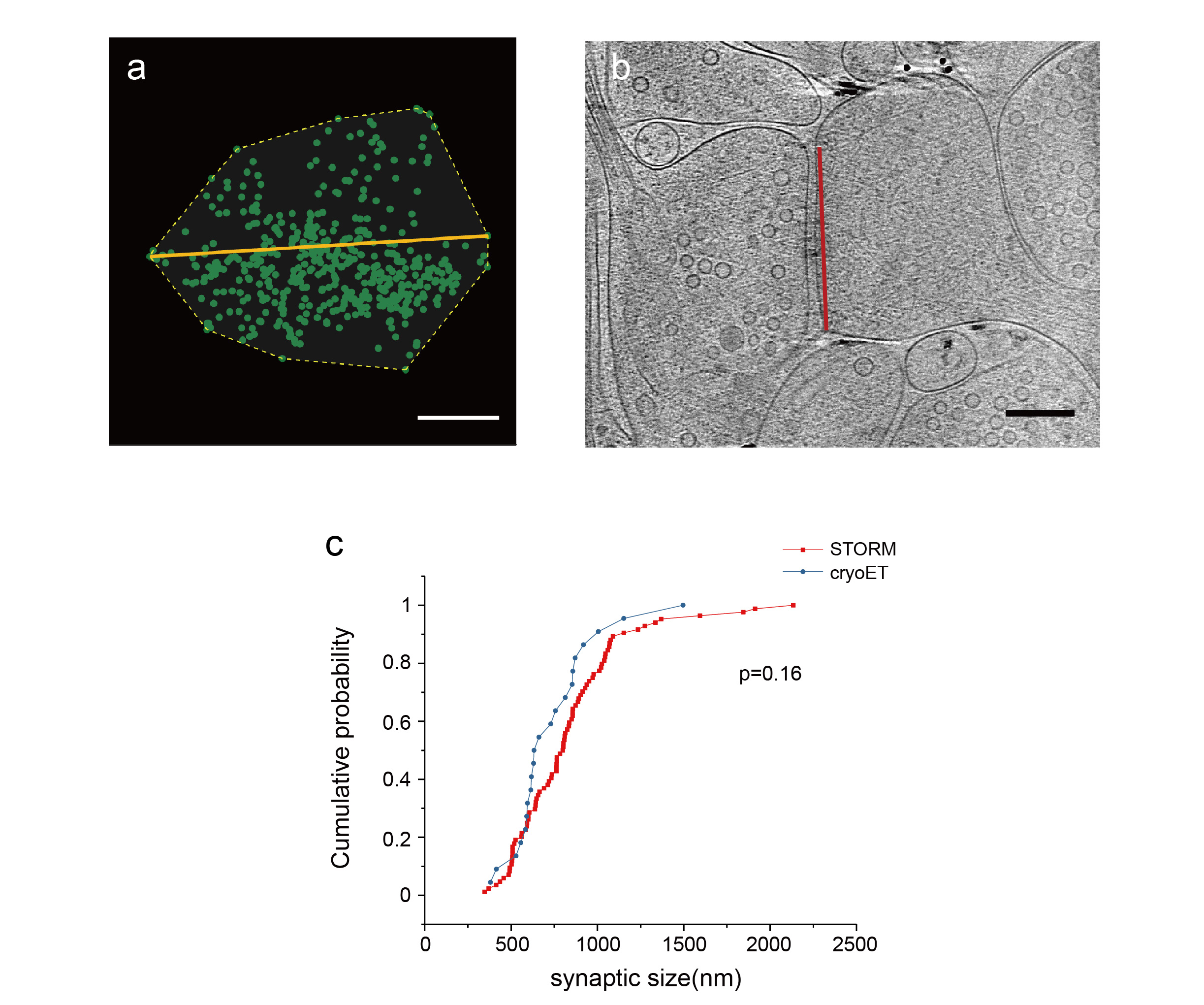


**Supplementary Fig. S9**. Size of spine synapses measured by STORM and cryo-electron tomography (cryoET).

**a** STORM image of GluN2B in a spine synapse. Yellow line indicates the size of the synapse measured by the maximal axis of the convex hull of GluN2B localizations (see Supplementary Fig S8). **b** A cross-section of the cryoET image of a spine synapse. Red line indicates size measurement of the synapse. **c** Cumulative histogram of size measurement of 84 spine synapses from STORM (red) and 21 spine synapses from cryoET (blue). The two distributions were not significantly different from each other (P=0.16, t test). Scale bar: 200nm.

**Supplementary Fig. S10**


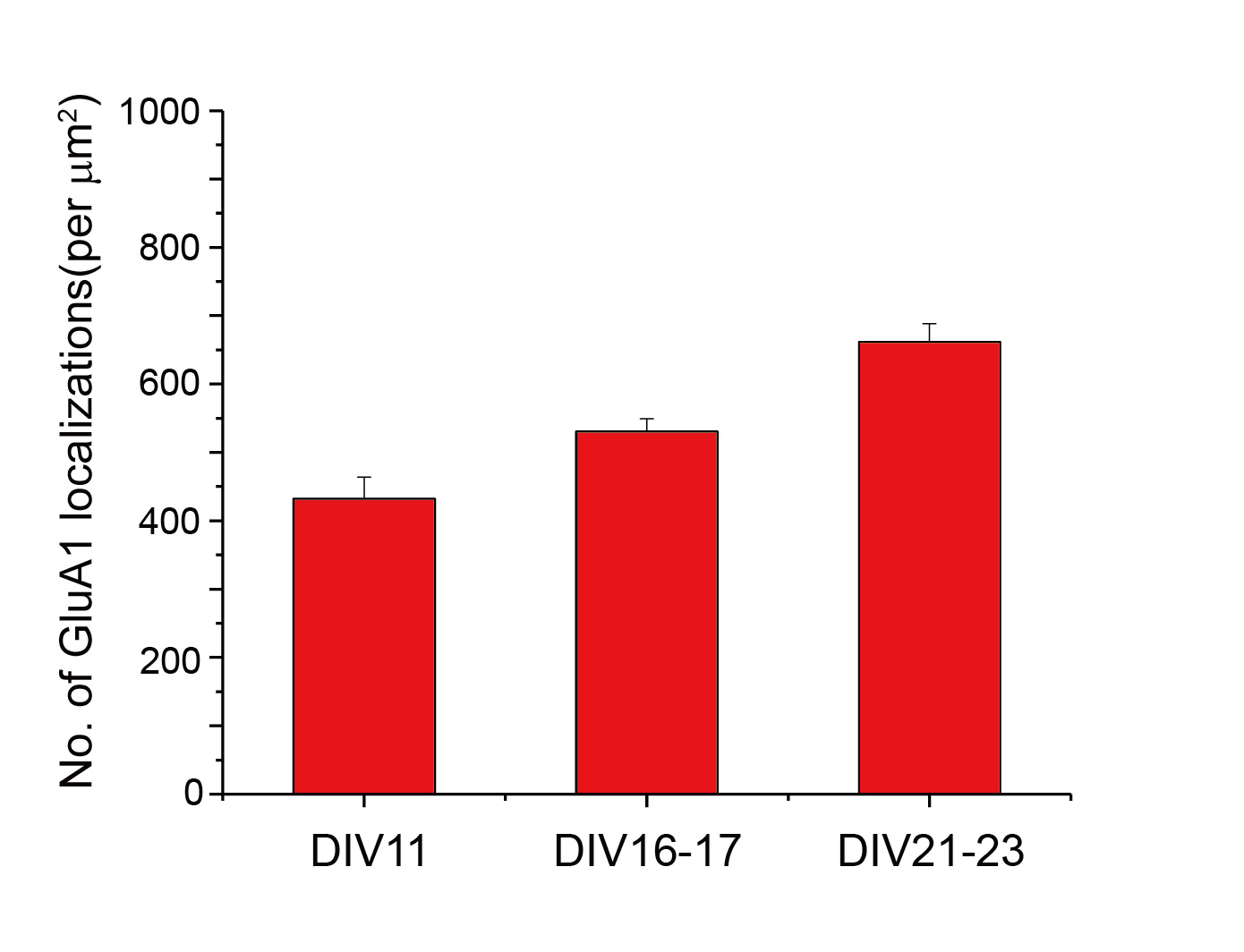


**Supplementary Fig. S10.** Expression of AMPARs in dendrite at different cultured stages.

Statistics of dendritic expression densities (localizations per μm^2^) of GluA1 at DIV11, DIV16-17, DIV21-23 respectively.

**Supplementary Fig. S11**


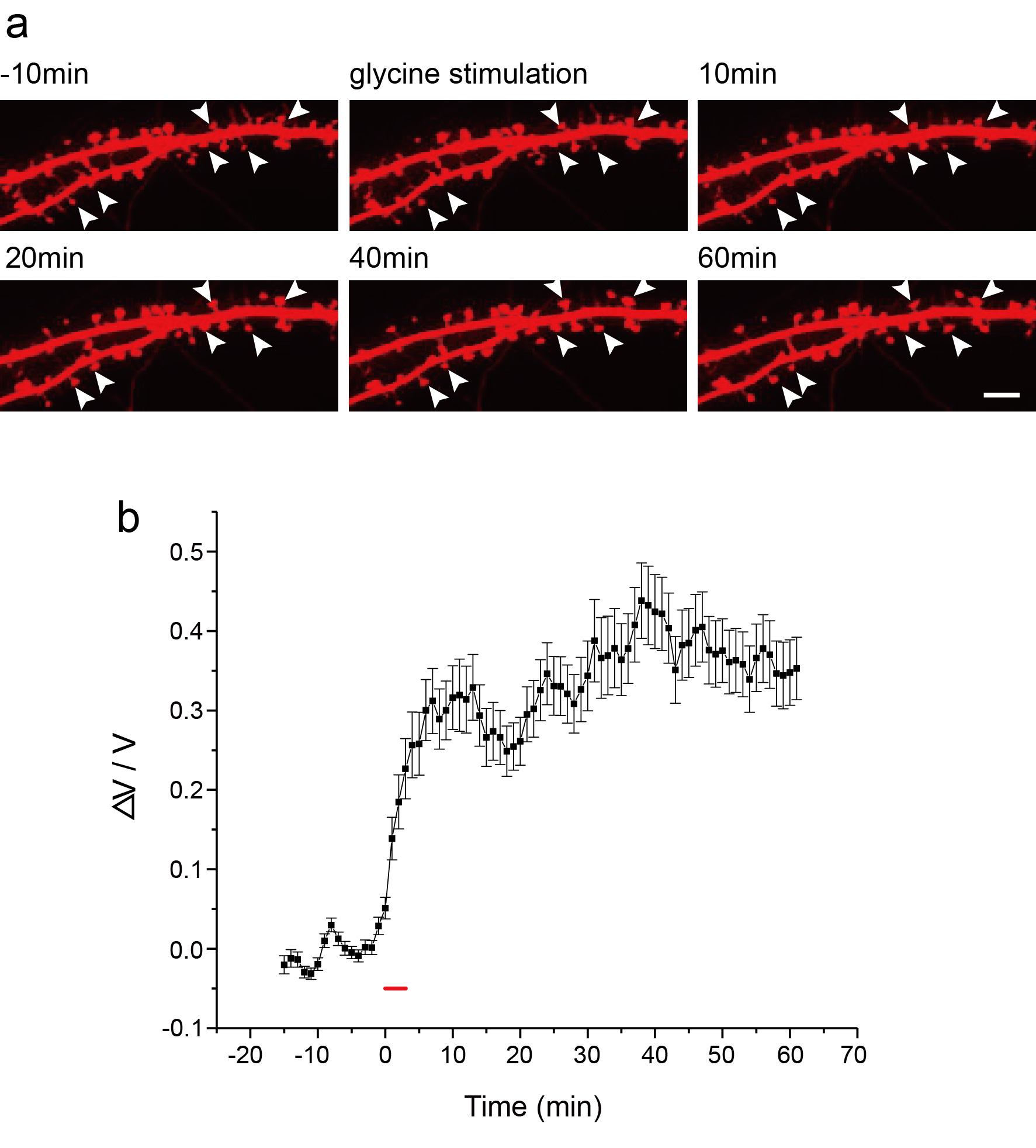


**Supplementary Fig. S11**. Synaptic enlargement during cLTP observed using live cell imaging.

**a** Time-lapse confocal images of dendritic spines from a DIV 17 neuron transfected with actin-mcherry at DIV10. Scale bar: 5μm. **b** summary of changes in synapse size following glycine simulation. ΔV / V is calculated by (I-I_baseline_)/I_baseline_. I is the maximal intensity of a synapse at a given time point, and I_baseline_ is average intensity during the baseline period. Red line indicates the time window of glycine stimulation.

**Supplementary Fig. S12**


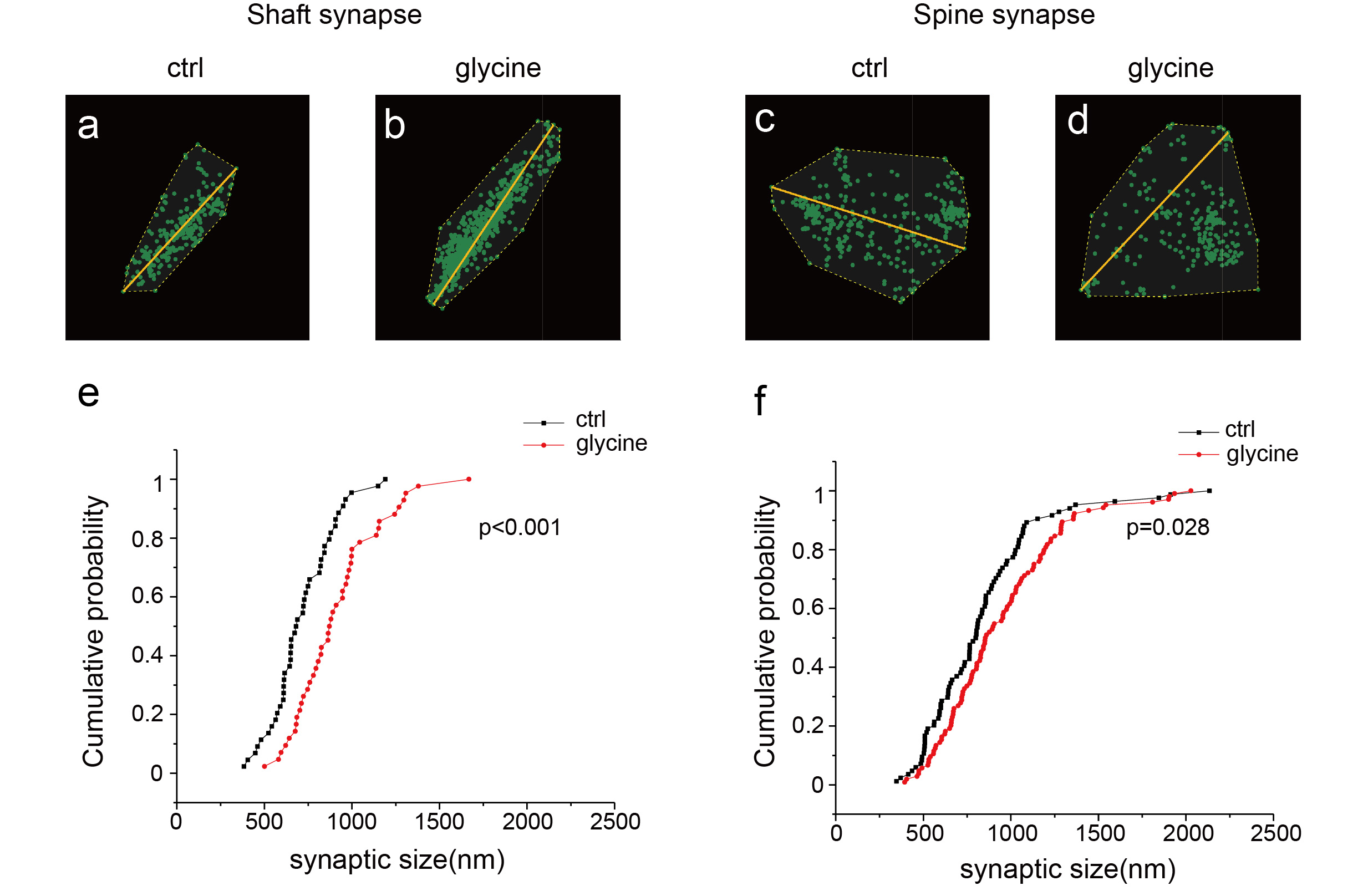


**Supplementary Fig. S12**. Changes in synaptic size after glycine stimulation.

**a-d** STORM image of synaptic GluN2B localizations in shaft **(a, b)** and spine **(c, d)** synapses in control **(a, c)** and glycine **(b, d)** groups. Scale bar: 200nm. Yellow line indicates synaptic size measured by the maximal axis of the convex hull of GluN2B localizations (see Supplementary Fig 8). **e-f** Cumulative histogram of the sizes of shaft **(e,** n=44 in control group and 42 in glycine group**)** and spine **(f,** n=84 in control group and 104 in glycine group**)** synapses in control (black) and glycine stimulated (red) group. The two distributions were significantly different from each other. P<0.001 in **e** and P=0.028 in **f**, t test.

**Supplementary Fig. S13**


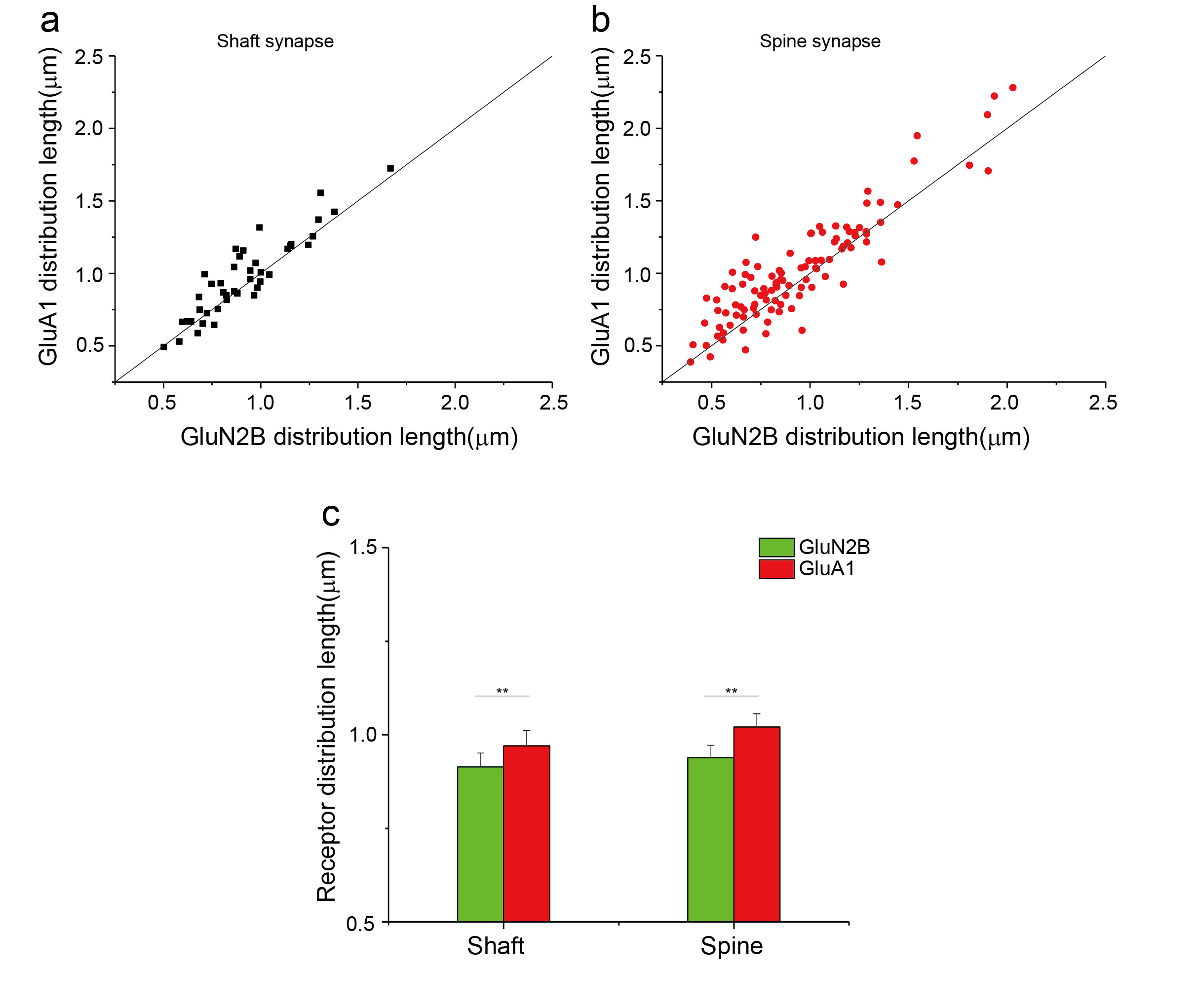


**Supplementary Fig. S13.** Spatial distribution of AMPARs and NMDARs in shaft and spine synapses after cLTP.

**a-b** Scatter plot of distribution lengths of GluN2B and GluA1 in shaft (**a**) and spine (**b**) synapses after cLTP. **c** Comparison of distribution lengths of synaptic GluN2B (green) and GluA1 (red) after cLTP. N=42(shaft), 104(spine). paired t-test.

**Supplementary movie legend**

**Supplementary movie. S1.** STORM localizations of GluA1 (red) and GluN2B (green) in a shaft synapse displayed in 3D view. Scale bar: 500nm.

**Supplementary movie. S2.** STORM localizations of GluA1 (red) and GluN2B (green) in a spine synapse displayed in 3D view. Scale bar: 500nm.
